# Evidence of Another Anthropic Impact on *Iguana delicatissima* from the Lesser Antilles: The Presence of Antibiotic Resistant Enterobacteria

**DOI:** 10.3390/antibiotics10080885

**Published:** 2021-07-21

**Authors:** Gustavo Di Lallo, Marco Maria D’Andrea, Samanta Sennati, Maria Cristina Thaller, Luciana Migliore, Gabriele Gentile

**Affiliations:** 1 Department of Biology, Tor Vergata University, 00133 Rome, Italy; dilallo@bio.uniroma2.it (G.D.L.); mcthaller@gmail.com (M.C.T.); luciana.migliore@uniroma2.it (L.M.); gabriele.gentile@uniroma2.it (G.G.); 2 Department of Medical Biotechnology, University of Sienna, 53100 Sienna, Italy; samantasennati@gmail.com; 3 e-Campus University, 22060 Novedrate, Italy

**Keywords:** critically endangered, IUCN Red List, action plan, Caribbean reptiles, *Escherichia coli*, squamata

## Abstract

The improper use of antibiotics by humans may promote the dissemination of resistance in wildlife. The persistence and spread of acquired antibiotic resistance and human-associated bacteria in the environment, while representing a threat to wildlife, can also be exploited as a tool to monitor the extent of human impact, particularly on endangered animal species. Hence, we investigated both the associated enterobacterial species and the presence of acquired resistance traits in the cloacal microbiota of the critically endangered lesser Antillean iguana (*Iguana delicatissima*), by comparing two separate populations living in similar climatic conditions but exposed to different anthropic pressures. A combination of techniques, including direct plating, DNA sequencing and antimicrobial susceptibility testing allowed us to characterize the dominant enterobacterial populations, the antibiotic resistant strains and their profiles. A higher frequency of *Escherichia coli* was found in the samples from the more anthropized site, where multi-drug resistant strains were also isolated. These results confirm how human-associated bacteria as well as their antibiotic-resistance determinants may be transferred to wildlife, which, in turn, may act as a reservoir of antibiotic resistance.

## 1. Introduction

The impact of the emergence and spread of antibiotic resistance in bacteria is now a widespread problem and a global concern [1]. Indeed, the extensive and often improper use of antibiotics has promoted the selection and dissemination of resistance in bacterial communities exposed to human activities [2]. The abundance and diversity of resistant bacteria and resistance genes in the environment (e.g., surface waters, soils and forests) is directly related to the impact of human activities [3]. Furthermore, even the diversity of the resistome (the amount/kind of resistance genes in a given environment) is linked to anthropic stress, since resistance to synthetic or semi-synthetic antibiotics that do not occur in nature abound in anthropized environments [4]. Many studies have highlighted the presence of acquired resistance traits in the commensal microbiota of various wildlife animals, mainly mammals and birds, in both promiscuous and remote environments (for an overview, see Pallecchi et al. [5]). However, wherever the proximity and/or promiscuity between man and wildlife is high, resistant bacterial isolates and/or resistance genetic determinants can circulate more easily in wild animals, thus, antibiotic resistant bacteria are likely observed in anthropogenic biased sites (for a review see Allen et al. [6]). The diffusion of resistant strains and/or resistance determinants related to antibiotic abuses in both husbandry and agricultural practices, have persuaded researchers to look mainly at animals as the reservoir for bacteria that potentially threaten human health (e.g., Woodward et al. [7]; Sylvester et al. [8]; Bautista-Trujillo et al. [9]). However, especially when endangered animal species are considered, the exchange of microorganisms caused by a high anthropic pressure should be assessed as a further element of risk and used as a tool to evaluate its extent. The presence of acquired antibiotic resistance and/or human associated bacteria, therefore, could provide a tool to monitor the extent of human exposure on some animal species [10,11].

**Figure 1:**
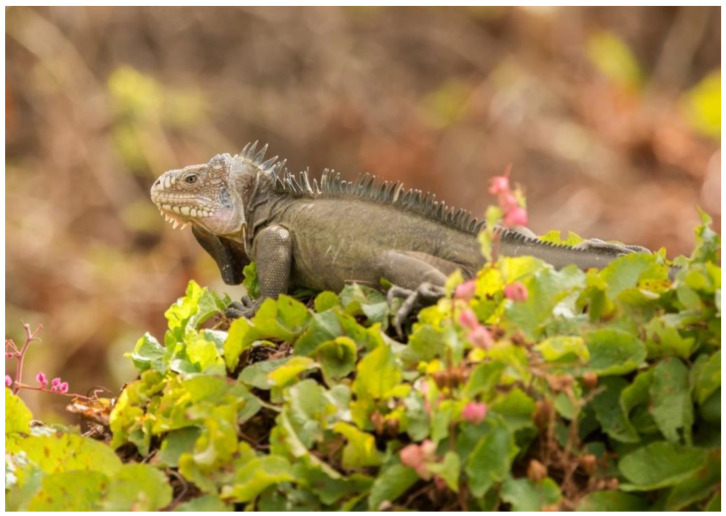
Adult male of *I. delicatissima* from St. Eustatius, Lesser Antilles (Photo: courtesy of Thijs van den Burg).

To this purpose, we have investigated both the nature of enterobacterial species and the possible presence of acquired resistance traits in the cloacal microbiota of the lesser Antillean iguana (*Iguana delicatissima*, endemic in the West Indies; Figure 1) by comparing two separate populations living in very similar climatic conditions, but exposed to dramatically different anthropic pressures. In particular, we focused on two islands: Saint Barthélemy (St. Barth) and Chancel Island, Martinique. In the first location, a wild population of *I. delicatissima*, is forced to have close contact with humans on a small island of 24 Km^2^, where a community of approximately 9000 human residents live, and tourism is active all year-round. In the last decades, the economic development of the island has increased rapidly, causing both fragmentation of the relevant iguana habitat and the dispersion of waste into the ground, with the subsequent pollution and contamination of phreatic waters and surface ponds. This situation is of concern and calls for a careful evaluation of the impact of this anthropic pressure on wild animals. Chancel, the second site of our study, is a very small (0.7 Km^2^) private island, characterized by similar climatic conditions but, differently from St. Barth, Chancel Island is inhabited by a single person. As in St. Barth, several domestic animal species have been introduced by the single resident of this island. Even though some of these individuals may be the result of local breeding over a number of generations, the turnover of domestic animals cannot be excluded.

Although *I. delicatissima* can swim for short distances, it is likely that the population from Chancel Island is quite isolated from Martinique despite the short geographic distance that separates Martinique and Chancel (approximately 0.3 Km). Migration from Martinique is not considered a driver of the increase in the census population in the Chancel Island, as observed from the estimated 250 individuals in 1994 [12] to more than 1000 in 2013 [13]. The increase is likely due to hunting control, an increase in tree cover, improvement and protection of nesting sites, and control of domesticated, free-roaming dogs [14]. There is no opportunity for migration between Chancel Island and St. Barth as the distance between the two islands exceeds 400 Km and cannot be covered either by active swimming or by passive transportation.

**Figure 2:**
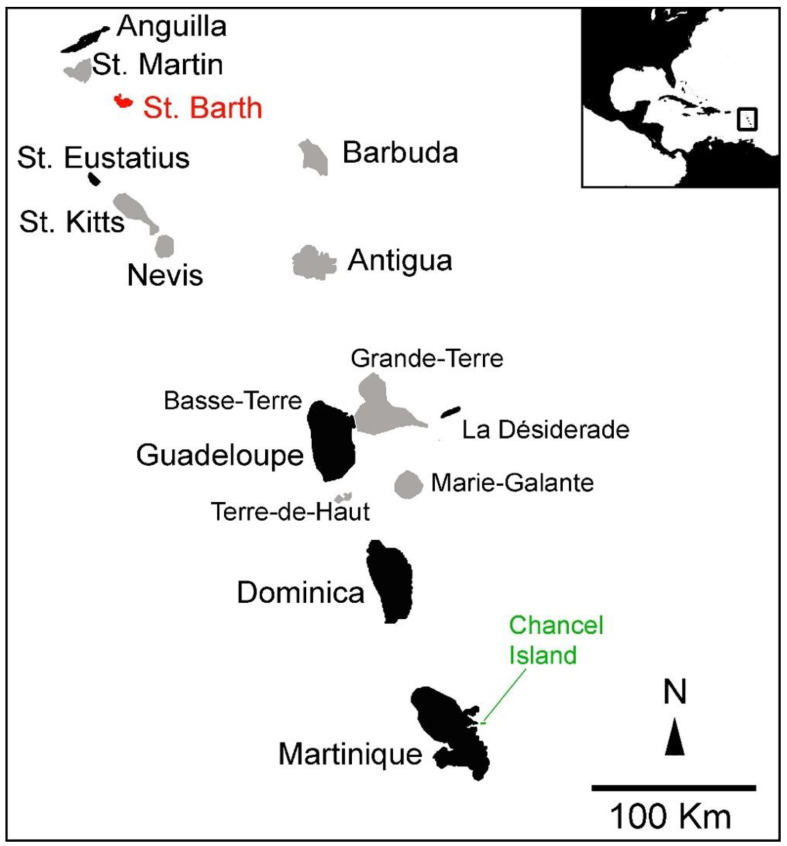
Distribution of *I. delicatissima* across the Lesser Antilles from the IUCN Red List of Threatened Species 2018 (redrawn, from van den Burg et al., 2018). Islands where the species occurs are shown in black. The grey colour indicates extinction in recent times. St. Barth is in red, while Chancel Island is in green.

Alien species impact both islands and could act as both reservoirs and vehicles of antibiotic resistance. Besides providing an excellent experimental framework for testing the efficacy of the transfer of enterobacterial communities and associated antibiotic resistance in wild species, *I. delicatissima* is also listed as critically endangered on the IUCN Red List [15]. The species once existed throughout the northern Lesser Antilles, but its presence is now limited to 774 Km^2^, if islands that have only pure Lesser Antillean iguana populations are considered (Figure 2). *I. delicatissima* is found in scrub woodlands, rainforests, and mangroves and shows good swimming ability in shallow waters. It is threatened by the invasive alien common green iguana (*I. iguana*), but it is also exposed to several other threats, including the direct impact of humans. One of the objectives of the 2014–2016 IUCN Action Plan for *I. delicatissima* [14] is to examine the influence of human populations on microbial diversity and antibiotic resistance in *I. delicatissima* throughout their range, in order to implement proactive conservation mediation programs.

## 2. Results and Discussion

### 2.1. Bacterial Isolates Identification

Of 98 swabs, 93 (43 from Chancel Island and 50 from St. Barth, hereafter, CHI and STB, respectively) yielded a confluent growth on the primary screening isolation plates and were further processed.

The dominant (DOM) enterobacterial population from the CHI samples consisted of 47 isolates; in four samples (9.3%) out of 43, two enterobacterial genus were present at high and comparable bacterial loads (*Enterobacter/Escherichia*, 2; *Enterobacter/Salmonella*, 1; *Escherichia/Salmonella,* 1). The isolates from CHI belonged to the *Citrobacter* (3; 6.4%), *Enterobacter* (14; 29.8%), *Escherichia* (8; 17.0%), *Klebsiella* (6; 12.7%), *Raoultella* (5; 10.6%) and *Salmonella* (11; 23.4%) genus. The presence of two enterobacterial species in the DOM of the STB site was observed in 23 (46.0%) out of 50 samples, hence, the total number of isolates was equal to 73. The identity of the DOM isolates at this site was as follows: *Citrobacter* (6; 8.2%), *Enterobacter* (12; 16.4%), *Escherichia* (24; 32.8%*), Klebsiella* (15; 20.5%), *Raoultella* (2; 2.7%), *Salmonella* (14; 19.2%).

All the recovered DOM isolates belonged to the family *Enterobacteriaceae sensu stricto*, as recently defined by Adeolu et al. [16]. Most isolates belonged to ubiquitous genera, usually recovered both from the environment and from the gut of animals and man (i.e., *Enterobacter*, *Citrobacter*, *Klebsiella* and *Raoultella*)*,* as well as being frequently isolated from human clinical samples [17]. *Salmonella* is quite common in the cloaca of many reptiles including iguanas [8,9,10,11,18], whilst *E. coli* is more commonly associated to the gut of warm-blooded animals and man, and are also used as an index of fecal contamination to assess water quality.

**Figure 3:**
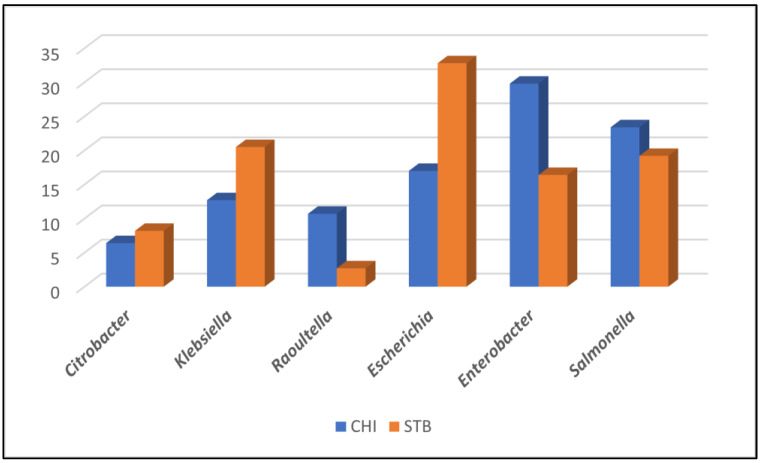
Isolation frequency (%) of the dominant enterobacterial genera obtained from cloacal swabs sampled at the Chancel Island (CHI) and Saint Barthélemy (STB) sites.

Interestingly, even though all enterobacterial dominant genera were found in both sampled sites, their abundance in the two localities was different, as testified by the Bray–Curtis similarity index, which was equal to 0.7. *Enterobacter* represented the dominant genus in the CHI samples (29.8%), whilst the main species in the samples from STB was *E. coli* (32.8%), possibly reflecting the higher anthropogenic activity at this site. As reptiles are not natural hosts for *E. coli*, the relatively high frequency of this species within the DOM isolates at CHI could be related to the presence of domestic animals. Finally, *Salmonella* and *Citrobacter* frequencies were similar in the samples set (Figure 3).

### 2.2. Resistance Traits in DOM Isolates

Excluding a few exceptions, the antibiotic susceptibility profile of the DOM isolates corresponded to that expected in wild type strains. Indeed, at the CHI site, a non-constitutive phenotype was observed in only 4 out of 47 DOM isolates, with resistance traits towards ampicillin (*E. coli*, 1); chloramphenicol (*Enterobacter,* 1); streptomycin only (*Salmonella*, 1) and streptomycin/tetracycline (*Enterobacter*, 1). Similarly, at the STB site acquired resistances were observed in only a few (7) isolates out of 73: one *E. coli* and one *S. enterica* isolate were found resistant to ampicillin, one *Citrobacter* of the *C. freundii* cluster was resistant to cefotaxime, two *S. enterica* and two *Enterobacter* spp. isolates were streptomycin resistant (Table 1). Collectively, these results suggest that resistance profiles observed in DOM isolates are more likely due to chromosomal mutations.

### 2.3. Resistance Traits in the Rare Antibiotic-Resistant (RAR) Isolates

A total of 76 RAR isolates were detected in the internal areas of the inhibition haloes: 20 from CHI and 56 from STB.

After discarding those isolates endowed with constitutive antibiotic resistance, there were 15 isolates with acquired resistance traits at the CHI site (*E. coli*, 8; *P. mirabilis,* 1; *Hafnia* spp., 1; *Serratia* spp., 1; *Enterobacter* spp., 4) and 29 at STB (*E. coli*, 19; *Klebsiella* spp., 5; *Enterobacter* spp., 4; *Raoultella* spp., 1).

The observed resistance traits are listed in Table 1 and Table 2. Although no resistance to carbapenems were observed, at the impacted STB site, several RAR isolates were resistant to antibiotics commonly employed for human clinical use, such as cefotaxime, gentamicin, amikacin and ciprofloxacin. On the contrary, these were totally absent in the RAR isolates from CHI. Moreover, the RAR isolates from CHI were endowed with a limited number of resistances, ranging from one to a maximum of three, whereas multiple antibiotic resistances were common among the RAR isolates from STB. Although the resistance profiles to β-lactams could be mostly associated to penicillinases, some isolates of *E. coli* (4), *Klebsiella* spp. (3) and *Raoultella* spp. (1) from STB showed a profile usually associated with the production of extended-spectrum β-lactamase (ESBL) enzymes [19].

The results of this study revealed a higher frequency of *E. coli* as the main enterobacterial species in the samples from STB when compared to the CHI control site. This could be regarded as an indication of the disturbance to the iguanas caused by the contamination of their environment by human activity. It should be noticed that the frequency of *E. coli* (32%) described in this work refers to the DOM species of the cloaca-associated enterobacteria, rather than to the simple presence of this bacterium in the samples, as in other studies, e.g., [8,9,10,11]. An even clearer indication comes from the comparison of antibiotic resistance frequency and profiles.

Antibiotic resistance profiles other than those expected in wild-type species has already been taken into account [10,11] as a possible index of anthropic pressure on endangered animal species, and some further criteria have been suggested to standardize and make the application of this trait easier in order to study the extent of contamination in both the environment and wildlife.

Apart from the mere presence of antibiotic resistant strains, Schwarz et al. [20] suggested to employ the occurrence of multi-drug resistant (MDR) strains, defined as those endowed of acquired resistance to at least three different antibiotic classes. Another possible index has been proposed by Bourély et al. [21], who demonstrated that in animal samples, the frequency of *E. coli* isolates endowed with a co-resistance to amoxicillin and tetracycline correlates with the total number of MDR *E. coli* isolates, being higher or comparable. The amplification of *tet* genes has been also proposed as a marker for water contamination [22].

In this study, no MDR strains were found among the RAR isolates from the control site (CHI), while at STB 16 out of 29 RAR (10 *E. coli*, 2 *Enterobacter aerogenes*, 3 *Klebsiella oxytoca* and 1 *Raoultella ornytinolytica*) matched this criterion.

The percentage of ampicillin and tetracycline resistant *E. coli* at STB (13 out 19 RAR *E. coli*, 68.4%) was higher than the percentage of MDR isolates (10 *E. coli* out 19, 52.6%). Thus, this marker clearly depicts the impacted condition at STB, while no one of such strains were found at the control CHI site.

The tetracycline resistant isolates belonging to naturally sensitive species were 1 *Klebsiella* spp. isolate from CHI and 15 isolates from STB (*E. coli*, 13; *Klebsiella* spp., 1 and *Raoultella* spp.,1). These isolates underwent a PCR screening targeting the *tetA* and *tetB* genes, which are largely diffused in *E. coli* from both human and animal sources [23,24]. A positive amplification reaction was obtained for 12 *E. coli* (*tetA*, 4; *tetB*, 3; *tetA* + *tetB*, 5), and the *Raoultella* spp. (*tetA*) isolate.

Due to its ubiquity and to the lack of intrinsic resistance, most studies have focussed on *E. coli* to facilitate the interpretation of resistance profiles, particularly against β-lactams. Indeed, in other species, resistance phenotypes to 3rd generation cephalosporins could possibly be due to hyper-production of intrinsic enzymes such as AmpC (*Enterobacter* and *C. freundii* cluster) or K1 (*K. oxytoca*) rather than to the presence of ESBL enzymes [19] being, therefore, difficult to interpret. Antibiotic resistance in *E. coli* isolates from *Iguana delicatissima* living in the Caribbean has been investigated recently within a large study [25]. Unfortunately, due to the different experimental approaches, the results of our study regarding the *E. coli* frequency within the DOM isolates could not be directly compared with those of Guoyomard-Rabenirina and colleagues [18]. Anyway, these authors did not find any antibiotic resistant *E. coli* in *I. delicatissima*, even though ESBL producing strains have been isolated from *Iguana iguana* living inside a hotel garden and in urban areas. These results reinforce the notion that anthropic activities pose a concrete risk of transfer of antibiotic resistant bacteria to wildlife.

Also in our experience, although MDR isolates belonging to other species have been found, most of those matching one of the above cited criteria were *E*. *coli* (Table 3). Therefore, even taking into account only *E. coli*, the frequency of MDR isolates, the percentage of ampicillin and tetracycline co-resistance and the incidence of genetic determinants for TetA and/or TetB, all point to both a highly impacted environment for the *Iguana delicatissima* population living in STB, and to the suitability of screening antibiotic susceptibility profiles as a tool for the survey of endangered animal species such as *I. delicatissima*. In addition, this investigation confirms the intimate relationship between the microbiota of humans and wildlife. Such a relationship may be finely tuned. It has been previously shown that if both the exposure to antibiotics and the anthropic pressure are negligible, acquired antibiotic resistance traits are not normally found in bacteria from wildlife, although suitable environmental conditions for bacteria circulation exist (*Conolophus pallidus* in Santa Fe Island; [10]). However, resistance can be found in bacteria from wildlife in the absence of antibiotic exposure (as a consequence of a reduced human presence) if wildlife is in close contact with domestic animals, as in CHI. When human density increases and wildlife is forced into promiscuity with both humans and domestic animals, as in STB, the number of resistances increases, as well as the number of antibiotics to which microbes show resistance. The resulting scenario generates concern for two reasons: (1) the use of antibiotics in human contexts may impact wildlife by introducing forms of resistance normally not present in the wild; (2) wildlife may act as a reservoir of man-derived antibiotic resistance, which may further impact human societies.

## 3. Materials and Methods

### 3.1. Sampling

A total of 98 *I. delicatissima* individuals were sampled, 47 at CHI and 51 at STB.

The samples were collected during two campaigns in January and April 2012. Cloacal samples were obtained from adult iguanas using sterile transport swabs with Cary Blair medium (Copan diagnostic, Brescia, Italy), and sent to the Biology Department of the Rome University, Tor Vergata, Italy, for microbiological analyses.

### 3.2. Bacterial Isolation and Identification

A direct plating method [26] was employed to detect both the DOM enterobacterial populations and the RAR isolates. Briefly, swabs were evenly spread onto MacConkey agar plates and antibiotic disks (Liofilchem s.r.l., Roseto degli Abruzzi, Teramo, Italy) were applied to the plates. After incubation at 37 °C for 18 h, the plates were inspected for growth and only samples yielding confluent or subconfluent growth were further processed, as described below. Antibiotic susceptibility results were interpreted according to the CLSI breakpoint for disk-diffusion testing. The presence of a substantial zone of growth inhibition indicated the overall susceptibility of all the populations in the sample. The absence or an inhibition halo with a very small diameter pointed to the resistance of at least the most represented bacterial populations in the sample. The presence of a clear inhibition halo, with isolated colonies inside, revealed the presence of RAR isolates.

The dominant species were detected by diluting a loopful of bacterial growth, taken from a confluent area well apart from the antibiotic disks, and plating on MacConkey agar to get isolated colonies.

RAR isolates were picked from the inside of the inhibition haloes and purified on Nutrient Agar plates (Liofilchem) supplemented with the same antibiotic, at the breakpoint concentration.

One colony for each observed morphology was picked, checked for purity by plating to isolated colonies on MacConkey agar, and stored for further analyses. The genus-level identification of the isolates was obtained by amplifying the 16S rRNA genes with the 341F (5′-CCTACGGGNGGCWGCAG-3′) and 805R (5′-GACTACHVGGGTATCTAATCC-3′) primers pair [27]. PCR amplicons were purified by using the Wizard PCR purification kit (Promega, Madison, WI) and then subjected to DNA sequencing on both strands at an external facility (Macrogen Inc., Seoul, Korea). The sequences were analyzed with the classifier tool at ribosomal database project site (http://rdp.cme.msu.edu/; last accessed on 2 July 2015). IMViC test (I = indole test; M = methyl red test; V = Voges-Proskauer test, iC= coliform ‘in Citrate’) together with the urease and malonate assay and EMB Levine agar plates (Liofilchem) were used to confirm the *E. coli* identification. The DOM isolates belonging to genera with species-specific wild type profiles, such as *Citrobacter* and *Proteus*, and all the RAR isolates were further identified at the species level by matrix-assisted laser desorption/ionization time-of-flight (MALDI-TOF) mass spectrometry by using the Vitek MS system (bioMérieux Inc.,Florence, Italy).

### 3.3. Antibiotic Susceptibility Profiles

The antibiotic-susceptibility profiles of both the DOM and the RAR isolates were determined with the Kirby–Bauer technique according to CLSI performance standards (CLSI M100). Ten antibiotics belonging to different classes were tested by using paper disks (Liofilchem) arranged on plates with a Thermo Scientific™ Oxoid™ antimicrobial susceptibility disc dispenser (Thermo Fisher Scientific™, Waltham, MA, USA): ampicillin (10 µg), streptomycin (10 µg), kanamycin (30 µg), gentamicin (10 µg), amikacin (30 µg), nalidixic acid (10 µg), ciprofloxacin (5 µg), chloramphenicol (30 µg), trimethoprim/sulfamethoxazole (1.25/23.75 µg) and tetracycline (30 µg). The ampicillin resistant strains were further tested with amoxicillin/clavulanic acid (20/10 µg), cefotaxime (30 µg), cefepime (30 µg) and imipenem (10 µg). Besides the intrinsic resistances listed in CLSI M100, tetracycline resistance in *Serratia marcescens* was regarded as a species-specific trait [28].

PCR amplifications to test for the presence of the *tetA* and *tetB* resistance determinants were performed according to Guardabassi et al. [29].

### 3.4. Similarity between Chancel Island and St. Barth

The similarity between DOM enterobacteria of CHI and STB was estimated by using the Bray–Curtis [30] similarity index for abundance data; it was applied only on dominant-isolate data. The similarity index was calculated by using the software PAST 3.0 [31].

## Figures and Tables

**Table 1:** Antibiotic susceptibility of dominant (DOM) and rare (RAR) enterobacterial isolates obtained from *Iguana delicatissima* cloacal swabs.

Site	Categorization	Identification	n° of Isolates	AMP	AUG	CTX	FEP	S	K	CN	AK	NA	CIP	C	SXT	TE
CHI	DOM	*Citrobacter* (*C. koseri* cluster)	1	WT												
		*Citrobacter* (*C. freundii* cluster)	2	WT	WT											
		*E. coli*	8	1												
		*Enterobacter*	14	WT	WT			1						1		1
		*Klebsiella*	6	WT												
		*Raoultella*	5	WT												
		*Salmonella*	11					1								
CHI	RAR	*Enterobacter*	4	WT	WT			1				1		1	2	
		*E. coli*	8	8	4							1			1	
		*Hafnia*	1	WT	WT										1	
		*Proteus mirabilis*	1	WT								1				WT
		*Klebsiella*	1	WT								1				1
STB	DOM	*Citrobacter* (*C. koseri* cluster)	2	WT												
		*Citrobacter* (*C. freundii* cluster)	4	WT	WT	1										
		*E. coli*	24	1												
		*Enterobacter*	12	WT	WT			1								
		*Klebsiella*	15	WT												
		*Raoultella*	2	2												
		*Salmonella*	14	1	1			3								
STB	RAR	*E. coli*	19	13	4		9	8	7	5	4	2	3	7	13	
		*Enterobacter*	4	WT	4		1	2	1	1	3	1		2		
		*Klebsiella*	5	5	3	2	4	1	3		3	3	1	4	1	
		*Raoultella*	1	1	1			1			1	1		1	1	

CHI: Chancel Island; STB: St. Barthélemy; DOM: Dominant enterobacteria; RAR: rare antibiotic resistant enterobacteria isolated within an antibiotic inhibition halo; AMP: ampicillin; AUG: amoxicillin/clavulanic acid; CTX: cefotaxime; FEP: cefepime; S: streptomycin; K: kanamycin; CN: gentamicin; AK: amikacin; NA: nalidixic acid; CIP: ciprofloxacin; C: chloramphenicol; SXT: trimethoprim /sulfamethoxazole; TE: tetracycline. WT: wild-type resistance trait.

**Table 2:** Acquired resistance profiles of the rare antibiotic resistant isolates.

Site	Isolates (n°)	Acquired Resistance
CHI	*E. coli* (1)	AMP/AUG
	*E. coli* (4)	AMP
	*E. coli* (1)	AMP/AUG/NAL
	*E. coli* (1)	AMP/AUG/SXT
	*Enterobacter cloacae* complex. (1)	ST
	*Enterobacter cloacae* complex (1)	C/NAL
	*Enterobacter cloacae* complex (2)	SXT
	*Hafnia alvei*	SXT
	*Klebsiella pneumoniae*	NAL/TE
	*Proteus mirabilis*	NA
STB	*E. aerogenes* (1)	ST/K/AK/NA/SXT/
	*E. aerogenes* (1)	K/CN/NA/SXT/
	*E. coli* (1)	AMP
	*E. coli* (1)	AMP/AUG/
	*E. coli* (1)	AMP/AUG/CTX/TE
	*E. coli* (1)	AMP/ST/K/
	*E. coli* (1)	AMP/TE
	*E. coli* (1)	AMP/AUG/CTX/ST/K/CN/AK/C
	*E. coli* (1)	AMP/AUG/NA/SXT/
	*E. coli* (1)	AMP/AUG/SXT/TE
	*E. coli* (1)	AMP/NA/TE
	*E. coli* (1)	AMP/SXT/TE
	*E. coli* (1)	AMP/AUG/CTX/NA/C/SXT/
	*E. coli* (1)	AMP/AUG/ST/K/CN/C/TE
	*E. coli* (1)	AMP/ST/K/CN/NA/C/SXT/TE
	*E. coli* (2)	AMP/AUG/TE
	*E. coli* (2)	AMP/AUG/ST/K/CN/AK/TE
	*E. coli* (2)	AMP/AUG/ST/K/CN/AK/SXT/TE
	*E. aerogenes* (1)	CTX
	*E. aerogenes* (1)	CTX/NA/CIP
	*K. oxytoca* (1)	AUG/SXT/
	*K. oxytoca* (1)	AUG/CTX/FEP/ST/K/NA/SXT/
	*K. oxytoca* (1)	AUG/CTX/ST/K/NA/SXT/
	*K. oxytoca* (1)	AUG/CTX/ST/K/CN/NA/C/SXT/
	*K. pneumoniae* (1)	AUG/ST/TE
	*R. ornythinolytica (1)*	AUG/CTX/K/NA/SXT/TE

Abbreviations are as in Table 1.

**Table 3:** Analysis of St. Barthélemy rare antibiotic resistant (RAR) isolates, according to different impact evaluation strategies.

RAR Isolates (n°)	MDR Isolates	Ampicillin and Tetracycline Resistance	*tetA*/*tetB*
*E. aerogenes* (4)	2	NA	NA
*E. coli* (19)	10	13	12
*K. oxytoca* (4)	3	NA	NA
*K. pneumoniae* (1)	NA	NA	0
*R. ornythinolytica* (1)	1	NA	1

MDR = Multi-Drug Resistant; NA = not applicable.

## Data Availability

The data presented in this study are available on request from the corresponding author.
